# Central Role of β-1,4-GalT-V in Cancer Signaling, Inflammation, and Other Disease-Centric Pathways

**DOI:** 10.3390/ijms25010483

**Published:** 2023-12-29

**Authors:** Subroto Chatterjee, Rebecca Yuan, Spriha Thapa, Resham Talwar

**Affiliations:** The Johns Hopkins Hospital, 1800 Orleans Street, Baltimore, MD 21287, USA

**Keywords:** β-1,4-Galactosyltransferase-V, lactosylceramide, cancer, inflammation, cardiovascular disease, angiogenesis, cell proliferation

## Abstract

UDP-Galactose: Glucosylceramide, β-1,4-Galactose transferase-V (β-1,4-GalT-V), is a member of a large glycosyltransferase family, primarily involved in the transfer of sugar residues from nucleotide sugars, such as galactose, glucose mannose, etc., to sugar constituents of glycosphingolipids and glycoproteins. For example, UDP-Galactose: Glucosylceramide, β-1,4-galactosyltransferase (β-1,4-GalT-V), transfers galactose to glucosylceramide to generate Lactosylceramide (LacCer), a bioactive “lipid second messenger” that can activate nicotinamide adenine dinucleotide phosphate(NADPH) oxidase (NOX-1) to produce superoxide’s (O_2_^−^) to activate several signaling pathways critical in regulating multiple phenotypes implicated in health and diseases. LacCer can also activate cytosolic phospholipase A-2 to produce eicosanoids and prostaglandins to induce inflammatory pathways. However, the lack of regulation of β-1,4-GalT-V contributes to critical phenotypes central to cancer and cardiovascular diseases, e.g., cell proliferation, migration, angiogenesis, phagocytosis, and apoptosis. Additionally, inflammation that accompanies β-1,4-GalT-V dysregulation accelerates the initiation and progression of cancer, cardiovascular diseases, as well as inflammation-centric diseases, like lupus erythematosus, chronic obstructive pulmonary disease (COPD), and inflammatory bowel diseases. An exciting development in this field of research arrived due to the recognition that the activation of β-1,4-GalT-V is a “pivotal” point of convergence for multiple signaling pathways initiated by physiologically relevant molecules, e.g., growth factors, oxidized-low density lipoprotein(ox- LDL), pro-inflammatory molecules, oxidative and sheer stress, diet, and cigarette smoking. Thus, dysregulation of these pathways may well contribute to cancer, heart disease, skin diseases, and several inflammation-centric diseases in experimental animal models of human diseases and in humans. These observations have been described under post-transcriptional modifications of β-1,4- GalT-V. On the other hand, we also point to the important role of β-1-4 GalT-V-mediated glycosylation in altering the formation of glycosylated precursor forms of proteins and their activation, e.g., β-1 integrin, wingless-related integration site (Wnt)/–β catenin, Frizzled-1, and Notch1. Such alterations in glycosylation may influence cell differentiation, angiogenesis, diminished basement membrane architecture, tissue remodeling, infiltrative growth, and metastasis in human colorectal cancers and breast cancer stem cells. We also discuss Online Mendelian Inheritance in Man (OMIM), which is a comprehensive database of human genes and genetic disorders used to provide information on the genetic basis of inherited diseases and traits and information about the molecular pathways and biological processes that underlie human physiology. We describe cancer genes interacting with the β-1,4-GalT-V gene and homologs generated by OMIM. In sum, we propose that β-1,4-GalT-V gene/protein serves as a “gateway” regulating several signal transduction pathways in oxidative stress and inflammation leading to cancer and other diseases, thus rationalizing further studies to better understand the genetic regulation and interaction of β-1,4-GalT-V with other genes. Novel therapies will hinge on biochemical analysis and characterization of β-1,4-GalT-V in patient-derived materials and animal models. And using β-1,4-GalT-V as a “bonafide drug target” to mitigate these diseases.

## 1. Introduction

### 1.1. β-1,4-GalT Family

**Figure 1:**
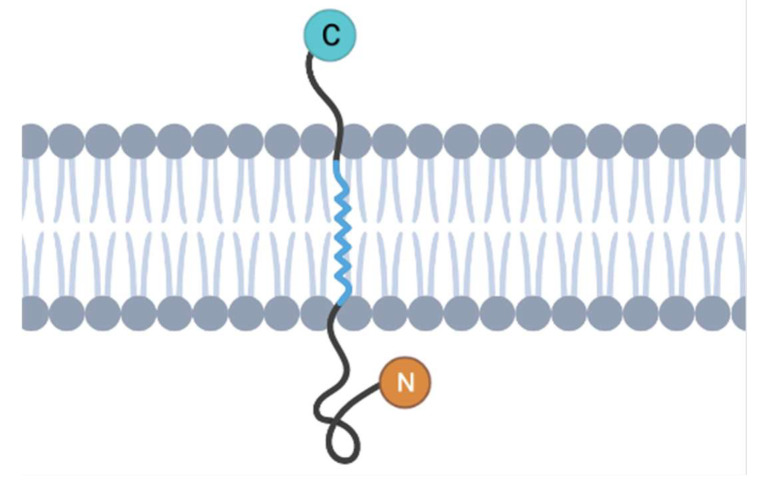
Hypothetical model of a β-1,4-GalT-V protein: a β-1,4-GalT-V protein is classified as a trans-Golgi glycosyltransferase (Glyco-T). It possesses a type II membrane protein structure consisting of a C-terminal catalytic domain oriented toward the lumen of the trans-Golgi and a brief N-terminal cytoplasmic segment [1].

β-1,4-GalTs are a family of glycosyltransferases, all having similar properties (i.e., they exclusively transfer galactose residues from a donor UDP-galactose via β-1,4 linkage to acceptor sugars, N-acetyl glucosamine (GlcNAc),glucose (Gl)c, and xylose(Xyl), which can be components of protein or lipids that have different functions). β-1,4-GalTs are single-chain transmembrane proteins primarily localized to the Golgi apparatus (Figure 1) [1]. The first mammalian β-1,4-GalT-I, the UDP-Gal: β-GlcNAc, was founded in 1986. It quickly evolved within a decade due to innovations in isolation, cloning, and expression of recombinant forms of glycosyltransferases, which catalyze the synthesis of complex oligosaccharides and glycoconjugates. The new strategy of identifying genes without the traditional isolation of proteins contributed to this advancement. Cloning strategies for glycosyltransferases were limited because the purification of many glycosyltransferases had problems with solubilization and stability. Thus, when purification to homogeneity was achieved, truncated forms of enzymes were isolated, showing that the enzyme activities must be a result of many different gene products. Transfection cloning strategies served as a breakthrough for access to the glycosyltransferase genes [2].

**Figure 2:**
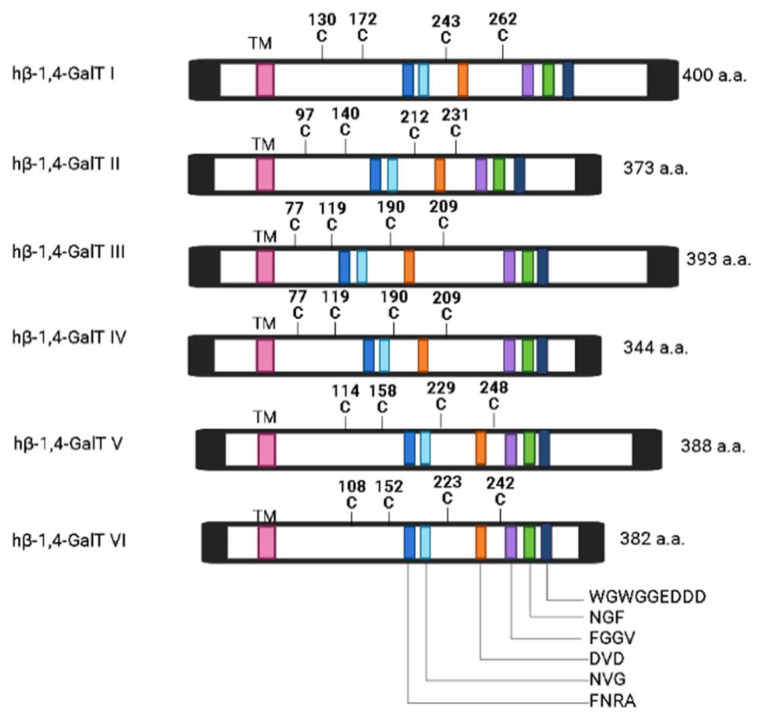
Homology of the β-1,4-GalT family genes. A sequence comparison of the human β-1,4-GalT family members indicates that although the binding site for the GlcNAc residue is highly conserved, the site that binds the extended sugar exhibits large variations. This is an indication that different β-1,4-Gal-T family members prefer different types of glycan acceptors with GlcNAc at their non-reducing ends [8]. The identification of specific binding sites and preferences among β-1,4-GalT family members provides opportunities for engineering these enzymes for targeted glycan modifications. This has practical applications in biotechnology, where precise control over glycosylation patterns is crucial for therapeutic protein production. The conservation of the GlcNAc binding site and the variability in the extended sugar binding site provide clues about the evolutionary history of the β-1,4-GalT family. Studying these evolutionary patterns can deepen our understanding of how these enzymes have adapted to perform specific functions over time and can transform. Note that only six out of seven of the β-1,4-GalT family genes are pictured in this figure (figure redrawn from [8] upon seeking approval from the publisher).

There are seven human β-1,4-GalT proteins known to date: β-1,4-GalTs I, II, III, IV, V, VI, and VII. (Figure 2) [3]. Studies showed that all the conserved sequence motifs among the seven β-1,4-GalT genes had a putative catalytic domain with predicted type II transmembrane topology with an N-terminal hydrophobic signal anchor sequence (Figure 1) [2]. Among seven β-1,4-GalTs, a putative transmembrane domain (TM, left), other amino acid sequences, and Cys-residues (C) are conserved (Figure 2). β-1,4-GalTs genes transcribe proteins composed of amino acid sequences ranging from 344 to 400 amino acids. They are expressed in various human tissues and mouse mammary glands. Additionally, β-1,4-GalTs are differentially expressed, with β-1,4-GalT-I and -V most differentially expressed [2]. The UDP-Gal binding domain was identified to be in the C-terminal region, between amino acid residues 341 and 351. Studies showed that β-1,4-GalT-I is the most efficient in galactosylating i/I structures of glycoproteins. Before the human genome was deciphered, β-1,4-GalT-II was known as LacCer synthase, which is important in glycosphingolipid (GSL)/LacCer biosynthesis [4,5]. Thereafter, it became apparent that there was another gene that had ~68% nucleotide sequence similarity with β-1,4-GalT-II. Accordingly, β-1,4-GalT-II was renamed as β-1,4-GalT-VI and the other gene as β-1,4-GalT-V. The latter11f1 enzyme is reported to be associated with the Golgi apparatus, cisterna, and Golgi membrane, as well as the cell membrane. The human β-1,4-GalT-V ortholog is located at 20q13.13. The optimum pH is 6.8, and the enzyme requires Mn^+2^, Mg^+2^, and detergent for maximum activity.

However, a controversy soon appeared in the literature stating that the acceptor substrate specificity for β-1,4-GalT-V indicated its potential in O-glycosylation of proteins but not GlcCer to generate LacCer. Using a Chinese hamster ovary mutant cell line (gift from Dr. Pamela Stanley, Albert Einstein College of Medicine) that exclusively produced β-1,4-GalT-V, we showed that these cells had LacCer synthase activity. Subsequently, Sato and Furukawa showed that, indeed, the β-1,4-GalT-V gene transcribes an enzyme involved in LacCer biosynthesis [6].

GSLs are located primarily, but not exclusively, in the outer leaflet of the plasma membrane. They can modify the activity of membrane receptors, such as those for insulin, epidermal growth factor, and nerve growth factor. Lactosylceramide may also be associated with the cell membrane but is readily transported to and stored in cytoplasmic vesicles in human neutrophils and human kidney proximal tubular cells. Furthermore, they are known to undergo remarkable changes during development, cellular differentiation, inflammation, and proliferation. This review presents significant contributions that provide context and functional information on the β-1,4-GalT-V gene/protein and its implications in angiogenesis, atherosclerosis, inflammation, cell proliferation, phagocytosis, and cancer pathways. Thus, our translational medicine research efforts focused on uncovering mechanistic links between β-1,4-GalT-V, and mediators of disease states. These efforts are aimed at developing novel diagnostics and therapeutics to improve the quality of life of healthy subjects and patients. In addition, we focus on improving our knowledge base for further research and development, as many outstanding reviews on other β-1,4-GalT transferases already exist in the field [7].

### 1.2. Unity in Diversity: The Involvement of β-1,4-GalT-V in Obesity, Cancer, Inflammation, and Heart Disease

We have shown the convergence of diverse critically and physiologically important molecules like oxidized LDL (implicated in atherosclerosis/obesity), epidermal growth factor (EGF) and vascular endothelial growth factor (VEGF) (implicated in cancer), and tumor necrosis factor-α (TNF-α) (implicated in inflammation) via activating β-1,4-GalT-V [9]. These observations may reflect upon a serious consequence of dysregulation of β-1,4-GalT-V in health and disease due to the following reasoning. First, data from the World Foundation of Obesity has predicted that by 2035, nearly 40% of the world’s population (approximately 4 billion people) will become obese. This will largely occur in young adults in Asia and Africa. Second, since obesity is closely associated with cancer (CRC) and heart disease, this will heavily weigh on the health and welfare of several nations [10]. Longitudinal studies on body mass index reveal that the COVID-19 pandemic may have contributed to a rapid increase in obesity among children aged 19 [11].

Colorectal cancer (CRC) is one of the leading diagnosed malignancies in the world, with more than 1.9 million new cases and 930 thousand deaths in 2020 [12]. Screening for CRC has proven to be difficult, with only 30–40% of patients diagnosed in Stage 1 or 2, despite being the third leading cancer in prevalence [13]. Part of this difficulty arises from the progressive nature of CRC and various epigenetic changes that accumulate tumor heterogeneity and tumor growth [14]. Based on 2023 data, CRC is 33% higher in males than females and 20% higher in African American populations than white populations [15]. Previous studies have also shown that inflammation accompanies obesity, atherosclerosis, and cancer, as well as the progression of these diseases. Since TNF-α can activate β-1,4-GalT-V, produce LacCer, and promote inflammation by way of the infiltration of neutrophils, β-1,4-GalT-V has a central role in multiple inflammatory diseases. Biochemical analysis and characterization of β-1,4-GalT-V in patient-derived materials and animal models of colorectal cancer (CRC) can help uncover β-1,4-GalT-V contribution to convergent pathways in inflammation and cancer and point to novel cancer therapies. Colorectal cancer (CRC) tumor cells have been shown to overexpress β-1,4-GalT-V compared to healthy/normal cells, releasing them into the body fluids. Thus, their detection has been suggested as a diagnosis/prognosis CRC biomarker. CRC is known to exhibit aberrant glycosylation and metabolism, leading to increased β-1,4-GalT-V levels [2,9].

## 2. Post-Transcriptional Modifications (PTM) of β-1-4GalT-V by Effector Molecules

**Figure 3:**
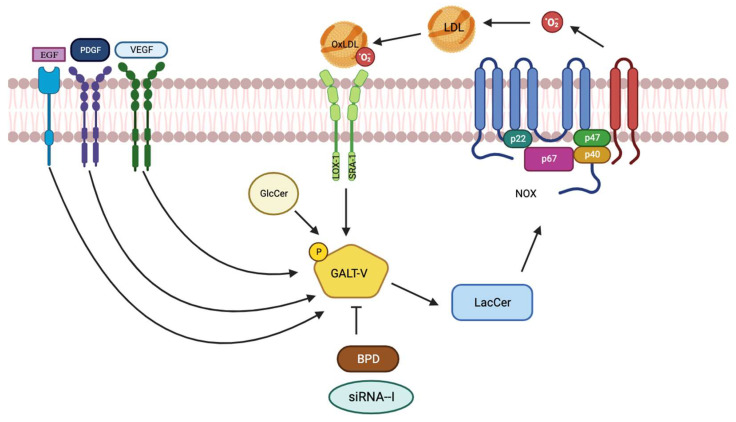
Biochemical pathway by which oxidized LDL phosphorylates/activates β-1,4-GalT-V, producing Lactosylceramide. In turn, LacCer activates NOX by facilitating its assembly with p67phox, p40phox, p47phox, and p22phox to generate superoxides (O_2_^−^). As superoxides are membrane-permeable, they cross the cell membrane and oxidize LDL to produce oxidized LDL. This cycle is mitigated by β-1,4-GalT-V gene ablation and/or use of a biopolymer-encapsulated GSL synthesis inhibitor, such as BPD.

It is important to understand the processes and regulation of β-1,4-GalT-V as LacCer (Figure 3); the product of LacCer synthase activity is a bioactive/bonafide lipid “second messenger” and is ubiquitously present in tissues. Furthermore, LacCer serves as a precursor to a large family of GSL, e.g., sulfatides, gangliosides, and complex neutral glycosphingolipids, as these homologs play important roles in regulating various phenotypes that affect disease pathophysiology. Most importantly, a statistically significant increase in the expression of the β-1,4-GalT-V gene in human CRC tissue, accompanied by a marked increase in β-1,4-GalT mass, LacCer synthase activity, and LacCer generation, has been reported recently [9]. However, the mechanisms by which the β-1,4-GalT-V gene is regulated in human CRC are not known. There are also over a hundred different ways by which PTM can occur in a protein that may alter function. Myristylation, palmitoylation, isoprenylation, farnesylation, acylation, amidation, glycation, ubiquitination, N- and O-linked glycosylation, OGlcNaCation, citrulination, and methylation are among more common PTMs reported thus far [16].

### 2.1. Phosphorylation

Pioneering studies relevant to mitogen-activated protein kinases (MAPK) constitute the kinase cascade in cell proliferation, as well as the activation of several oncogenes like Ras/Raf/extracellular signal-regulated kinase (ERK) and Akt signaling pathways [17]. Herein, phosphorylation was associated with the activation of these enzymes [18]. While the LDL receptor was essential to decrease the activity of LacCer synthase in cultured human kidney proximal tubular cells, even with a lack of LDL receptors, e.g., in familial hypercholesterolemic homozygous patients, LDL stimulated the activity of this enzyme. Moreover, methylated LDL also increased the activity of LacCer synthase [9]. This suggests that methylated LDL and/or LDL upon oxidation entered the cells via an LDL receptor-independent pathway or “scavenger pathway” through the receptors LOX-1 and SRA-1. (Figure 3)

Although treatment of human arterial smooth muscle cells with oxidized LDL stimulated β-1,4-GalT-V activity, ultimately leading to cell proliferation, the mechanism of β-1,4-GalT-V activation was not known. To examine if phosphorylation is involved, first, cells were tagged with 32P, followed by incubation with oxidized LDL. Next, β-1,4-GalT-V was immune-precipitated and hydrolyzed, amino acids were separated by two-dimensional chromatography, and radio-autographic images were recorded. These studies revealed that oxidized LDL phosphorylated serine, threonine, and tryptophan residues in β-1,4-GalT-V in a time- and concentration-dependent manner. In contrast, when cells were pre-incubated with D-PDMP, another β-1,4-GalT-V inhibitor, it blocked oxidized LDL-induced phosphorylation of these amino acids as well as enzymatic activity. Furthermore, treatment blocked the oxidized LDL-induced increase in LacCer levels, the oncogenes above, MAPK, and ultimately, cell proliferation [19]. Furthermore, 1-palmitoyl-2-(5-oxovaleroyl)-sn-glycero-4-phosphocholine (POVPC) found in human minimally oxidized LDL was identified as a biologically active component pivotal in arterial smooth cell proliferation via recruiting β-1,4-GalT-V, LacCer, and the phosphokinase pathway [20]. Together, these studies suggest that in normal cells, LDL receptors decrease LacCer synthase activity to control LacCer generation and cell proliferation. In contrast, oxidized LDL enters cells via an LDL receptor-independent pathway, and the phosphorylation of β-1,4-GalT-V is essential for activation and downstream oncogenic signaling pathways and hyper-proliferation in cardiovascular disease.

In addition to oxidized LDL described above (Figure 3), many critically and physiologically relevant molecules, e.g., growth factors, have been shown to activate β-1,4-GalT-V and produce LacCer. In turn, LacCer activates downstream signaling pathways, ultimately affecting/upregulating cell-specific phenotypes. In all cases, these growth factors bind to their cognate receptors localized on the cell surface and subsequently activate β-1,4-GalT-V. For example, studies show that β-1,4-GalT-V is the major LacCer synthase in cultured human umbilical vein endothelial cells. Additionally, human arterial endothelial cells prepared from patients with colorectal cancer tumors exhibited a marked increase in the β-1,4-GalT-V gene expression but not β-1,4-GalT-VI. Thus, when human umbilical vein endothelial cells and/or human arterial endothelial cells were treated with VEGF, it significantly increased β-1,4-GalT-V activity to generate LacCer (Figure 3). Downstream, LacCer, via the activation of the phosphokinase pathway and NfkB, increased the expression of a major endothelial cell adhesion and cell junctional protein, platelet endothelial cell adhesion molecule, and angiogenesis [9].

Another agonist of β-1,4-GalT-V is platelet-derived growth factor (PDGF), which exerts a time- and concentration-dependent increase in activity in CHO mutant cells exclusively expressing the β-1,4-GalT-V gene/protein. LacCer thus generated activated NADPH oxidase (Nox-1) activity, producing superoxide, MAPK activation, and, eventually, cell proliferation (Figure 3). Similar studies using EGF, a major growth factor in malignancies, also recruited the β-1,4-GalT-V and LacCer pathway to contribute to cell proliferation [9].

### 2.2. Methylation of DNA—A Common Epigenetic Change in CRC

Epigenetic changes refer to stable changes in cell function without involving any alterations in the DNA sequence. One of the classical epigenetic changes in DNA is the methylation of its CpG islands. Such changes regulate the expression of genes via transcriptional regulation of corresponding genes. For example, smokers have less DNA methylation compared to non-smokers. Interestingly, when smokers quit smoking, this results in increased DNA methylation. Evidence has been put forward that aberrant methylation of the β-1,4-GalT-I gene is associated with CRC [14]. Also, DNA testing of stool samples for aberrantly methylated NDRG4 promoter and KRAS and BMP3 promoter regions [21] currently serves as a standard diagnostic measure for CRC. Sato and Furukawa have investigated how β-1,4-GalT-V is transcriptionally regulated in cancer cells [22]. They cloned the 2.3 kb 5′ flanking region in the β-1,4-GalT-V gene and determined that promoter activity was associated with the region 116/18, Transcriptional factors Ap2, Ap4, NMyc, Sp1, and an upstream stimulatory factor were found to bind to this promoter region 2′3′.

### 2.3. Sp1 a Transcriptional Regulator of β-1,4-GalT-V

β-1,4-GalT-V is a protein derived from a single gene product, so one important regulator of β-1,4-GalT-V is the β-1,4-GalT-V gene, as it controls expression. It has been shown that β-1,4-GalT-V’s mRNA transcript levels have been shown to increase 2–3-fold upon malignant transformation, which has implications in many cancer pathways [23]. In addition, the expression pattern of β-1,4-GalT genes changes in malignant transformation of cells despite little changes in enzymatic activity. This demonstrates that the function of the β-1,4-GalT-V gene is a crucial agonist/antagonist of the protein function.

Sp1 was originally found to be a transcription factor that binds to the GC box to activate transcription. In addition, the activation of genes by Sp1 is enhanced if there are multiple Sp1 binding sites on the gene. Since there are four binding sites of Sp1 on the β-1,4-GalT-V, and Sp1 was shown to bind to the promoter region, it has been demonstrated to be an important molecule that activates the promoter of the β-1,4-GalT-V gene. This study was the first to show that Sp1 transcriptionally regulated β-1,4-GalT-V, especially in cancer cells. Reducing Sp1 expression could be a possible step to suppressing tumor growth and metastasis through downregulation of the β-1,4-GalT-V gene [24].

More specifically, within the promoter region of the β-1,4-GalT-V gene, four Sp1, one N-Myc, one USF, one AP2, and one AP4 binding site(s) were identified, with Sp1 having the most prominent role in transcriptional activation. The β-1,4-GalT V gene binds to Sp1, which is a transcription factor that binds to the GC box to activate transcription, and mutations to the Sp1 binding site impair promoter activity. The Sp1 binding site localized at nucleotide position 81/69 was associated with promoter activity as mutations induced in the Sp1 binding site resulted in a loss in promoter activity. Conversely, transfection of a lung carcinoma cell line (A549) with Sp1cDNA increased promoter activity. Additionally, Mithramycin, which inhibits Sp1 binding, also blocked β-1,4-GalT-V gene expression and reduced promoter activation.

Later, it was also shown by Sato and Furukawa that gene expression or β-1,4-GalT-V is also regulated by the transcription factor Ets-1 [22]. Furthermore, Ets-1 was found to enhance promoter activity of Sp1 by binding to its binding site overlapping with Sp1′s binding site. Thus, Ets-1 is critical for transcriptional activation in angiogenesis, invasion, and cancer progression. In sum, β-1,4-GalT-V gene transcription is regulated by Sp1, and the loss of regulation by Sp1 may contribute to aberrant cell proliferation and cancer [25, 26, 27]. However, this tenet has not been addressed in the context of human CRC.

### 2.4. Role of β-1,4-GalT-V in Glycosylation—A Predominant Form of Post-Translational Modification

Glycosylation of newly synthesized proteins is among the most common post-translational/epigenetic modifications in mammals and has important implications for protein activity, unfolding, degradation, and cell functioning, as well as in cancer [28]. Glycosylation disorders and Golgi disorders affect N-glycan processing and include defects in glycosyltransferases, nucleotide sugar transporters, vacuolar pH regulators, and multiple cytoplasmic proteins that traffic glycosylation machinery within the cell and maintain Golgi homeostasis. Most of these processes are required for multiple glycosylation pathways. Defects occur in (1) the activation, presentation, and transport of sugar precursors; (2) in glycosidases and glycosyltransferases; and (3) in proteins that traffic glycosylation machinery or maintain Golgi homeostasis. Some defects strike only a single glycosylation pathway, whereas others impact several. Most glycosyltransferases are localized with the Golgi apparatus but can also be present with other cellular membranes and the cytoplasm, wherein glycosylation ensues, and glycosylation determines the further transport of the glycosylated molecule. Moreover, glycosylation determines the further transport of lipids and proteins and mediates whether the glycosylated protein will be secreted out of the cell or become an integral component of the cell membrane, e.g., the LDL receptor. And serum albumin, the major circulating protein in blood, is glycosylated in patients with diabetes due to an underlying modification factor that contributes to the alteration of albumin’s multitude of functions and the patients’ diabetic state.

**Figure 4:**
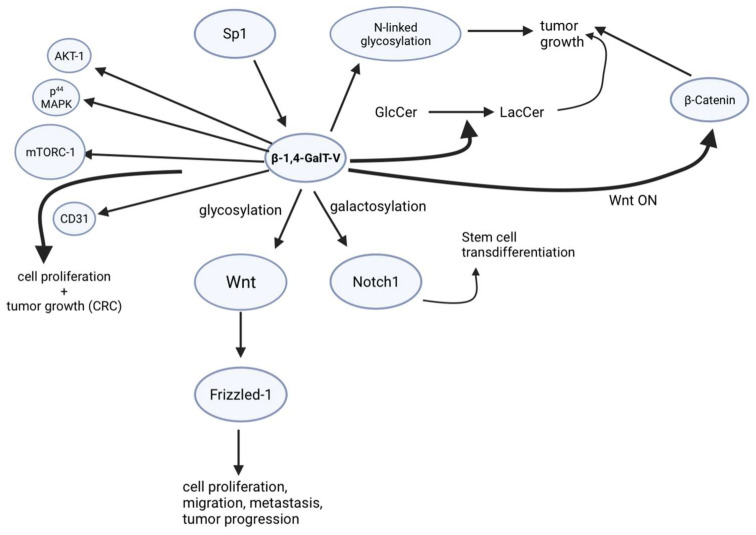
Signaling pathways by which β-1,4-GalT-V regulate various effector molecules in CRC.

One of the major functions of β-1,4-GalT-V is post-translational modifications, such as galactosylation and biosynthesis of N-linked oligosaccharides (Figure 4). This is important in cancer pathways because a key feature of the malignant transformation of cancerous cells includes an increased number of N-linked oligosaccharides. It was identified that reducing β-1,4-GalT-V expression levels decreased galactosylation of highly branched N-glycans, which are highly involved in cancerous growth and metastasis [3] More specifically, β-1,4-GalT-V is involved in glycosylating glucosylceramide and GlcNAc β-1-6 mannose group of the highly branched N-glycans, the mentioned hallmark of cancer cells [16]. It was found that expression of β-1,4-GalT-I is reduced in tumor tissues [9], and β-1,4-GalT-II is also demonstrated to decrease in transformed cells, altering glycosylation of the tumor. However, additional findings later revealed that there was an increase in gene expression of β-1,4-GalT-V by 2.25-fold in these CRC samples. When HCT-116 cells were treated with an inhibitor of glycosyltransferase, Phenyl-2-decanoylamino-3-N-morpholino-1-propanol (D-PDMP), there was a time- and dose-dependent decrease in cell proliferation through the mechanism of inhibiting GSL synthesis [13]. These findings show that glycosylation is an important modification in cancerous cells, and β-1,4-GalT-V plays a critical role.

Furthermore, altered N-glycosylation also results in structural and functional changes that affect integrin-mediated interactions and contributes to a more invasive or metastatic phenotype in colorectal cancers. The characterization of these mature αβ integrin subunits expressed in colorectal carcinomas revealed a smaller β chain in addition to the mature β-1 chain present in normal mucosa of the colon. The smaller β chain is present in the isolated cell membranes of colorectal cancers and comigrates with a diminished glycosylated precursor form of the mature β-1 integrin subunit found in healthy cells. Upon the malignant transformation of cells, the expression of the β-1,4-GalT-V gene increases in accordance with the increase in the amounts of highly branched N-glycan. Furthermore, inhibition of N-glycan processing in human colon adenocarcinoma HT-29 cells increased affinity to Type IV collagenase. This affinity contributes to diminished basement membrane architecture and more invasive or metastatic phenotype in colorectal cancer. As a result, the state of glycosylation plays a crucial part in the complex process that glycosylation plays during tissue remodeling and cancer progression. This points to the role of β-1,4-GalT-V in forming the diminished glycosylated precursor form of the mature β-1 integrin subunit that influences infiltrative growth and metastasis in human colorectal cancers. Novel therapies will hinge on biochemical analysis and characterization of β-1,4-GalT-V in patient-derived materials and animal models [29].

A key feature of malignant transformation of cells in cancer is an increased number of N-linked oligosaccharides. Previous studies have shown that the growth rates and metastatic properties of cell transformants are related to the number of N-linked oligosaccharides being synthesized by N-acetylglucosaminyl-transferase V (GlcNAcT-V), whose activities were reported to increase in malignantly transformed cells. Initially, β-1,4-GalTs were shown to be involved in the in vivo biosynthesis of N-linked oligosaccharides when examining the expression levels of β-1,4-GalT transcript in NIH3T3 and MTA, its malignant transformant [30]. Studies indicate that β-1,4-GalT-V is involved in the galactosylation of highly branched oligosaccharides characteristic of malignantly transformed cells [31].

In contrast, the expression of β-1,4-GalT-I was reduced in tumor tissues [7], and β-1,4-GalT-II was also demonstrated to decrease in transformed cells, altering glycosylation of the tumor. However, additional findings later reveal that there is an increase in gene expression of β-1,4-GalT-V indicating expression of highly branching N-link oligosaccharides in tumor cells, which provide support for the transferase involvement in altered glycosylation. Furthermore, in another study, the gene expression patterns of β-1,4-GalT-V are shown to drastically change without converting the β-1,4-GalTs’ activities in the malignant transformation of NIH3T3 [30]. Thus, it has been identified that β-1,4-GalT-V is partially responsible for glycosylation, where it has been found that β-1,4-GalT-V effectively glycosylates GlcCer and GlcNAc B-1-6 mannose groups of highly branched N-glycans, which is a characteristic of tumor cells [13].

In addition, increasing evidence has shown that aberrant glycosylation of cell surface receptors in breast cancer stem cells (BSCSs) results in oncogenic transformation, with the most frequent attributed to abnormal expression of glycosyltransferases. BCSCs are regulated by several signaling pathways, like Notch and Wnt/β -catenin, among others, which are synonymous with what has been previously discussed in other cancers. This is relevant as breast cancer is one of the most malignant cancers among women worldwide, with over 2 million new cases yearly and 600,000 deaths in 2018. As a type II membrane-bound glycoprotein mostly located in the Golgi, the β-1,4-GalT family is considered a therapeutic target in tumor progression and metastasis, which is still a large struggle in cancer treatment of this type. Of the seven members of the β-1,4-GalT gene family, β-1,4-GalT-V is strongly suggested as modulating the stem-ness of BCSCs and is upregulated by glycosylating the receptor Frizzled-1 and constantly activating Wnt/β-catenin signaling (Figure 4).

This is an important start in the connection of β-1,4-GalT-V in various pathways in cancer. In breast cancer, β-catenin has been shown to be a useful prognostic marker. In colon cancer, β-catenin has been found to interact with and inhibit NF-kβ, resulting in a reduction of NF-kB DNA binding, transactivation activity, and target gene expression. In hepatocellular carcinoma, β-catenin is known to be involved in various stages of hepato-carcinogenesis (Figure 4). In gastric cancer, increased β-catenin mRNA levels and mutational alterations of the APC and β-catenin gene have been demonstrated. In addition, β-catenin has been found to be physically complexed with T-cell factor family transcription factors and activate the transcription of several critical genes involved in cell proliferation, such as Wnt/Wingless signaling [32,33]. Transcriptionally inert T-cell factors become potent trans activators upon interaction with the Wnt signaling product β-catenin. Recently, Tcf factors have been reported as tumor inducers, aberrantly activating their target genes because of elevated β-catenin levels in many types of cancer. These abnormal β-catenin levels are usually caused by stabilizing mutations in β-catenin itself or truncating mutations in the adenomatous polyposis coli (APC) tumor suppressor gene. While this study was primarily focused on the breast cancer pathways of β-1,4-GalT-V and β-catenin, these are provocative results that may be an area of interest in other cancers, like colorectal cancer [34].

#### 2.4.1. Inhibitors of β-1,4-GalT-V

Currently, there are no known specific inhibitors for β-1,4-GalT-V. D-PDMP was chemically synthesized to inhibit the activity of UDP-Glucose β-1,4 glucosyltransferase, which transfers glucose to ceramide to generate glucosyl ceramide. However, we observed that in cultured human kidney proximal tubular cells and purified LacCer synthase from human kidney, D-PDMP also inhibited the activity of LacCer synthase and reduced cell proliferation. However, our studies in experimental animal models of human diseases, e.g., an ApoE^−/−^ mouse model of atherosclerosis [35], type II diabetic mice *(db/db)* [36], and other animal models by other investigators described throughout this review, have shown that D-PDMP decreases LacCer synthase activity. Thus, D-PDMP is now widely used as an inhibitor of LacCer synthase. This is largely due to its easy availability and the fact that it is well tolerated by experimental animals. For example, we fed D-PDMP to ApoE^−/−^ mice daily at 100 times the effective dose by oral gavage for up to 6 months without any effects on body weight or organ weight. In fact, treatment with D-PDMP increased hair growth and prevented hair greying and skin wounding in these mice fed a Western diet high in fat and cholesterol. In contrast, in untreated mice fed a diet high in fat and cholesterol, their hair had turned from black to grey and there was hair loss and numerous skin wounds.

We have also encapsulated D-PDMP within a biopolymer composed of polyethylene glycol and sebacic acid. This compound, called biopolymer-encapsulated D-PDMP or BPD, is more than 10 times more efficient than native D-PDMP in preventing cardiac hypertrophy [37]. This is because the transit time from oral delivery to the appearance in the gut is reduced significantly. Consequently, the T_1/2_ of 1 hr. by D-PDMP was increased to 48 hrs. in the case of BPD [37]. A targeted genetics approach to mitigate β-1,4-GalT-V gene protein expression using shRNA for β-1,4-GalT-V has been used successfully to mitigate VEGF-induced angiogenesis in cultured human umbilical vein endothelial cells [9] and there was a reduction in tumor growth in a mouse model of glioblastoma [26].

#### 2.4.2. Angiogenesis and β-1,4-GalT-V

Angiogenesis is the formation of new blood vessels from existing ones. It is an important physiological process that is required for embryonic development, vasculature formation, wound healing, and tumor growth. Therefore, lack of angiogenesis regulation may lead to various inflammatory diseases and cancer progression. Angiogenesis is mainly driven by VEGF, the most common growth factor critically needed for angiogenesis, which also plays a role in collateral blood vessel formation in diabetes and atherosclerosis [9]. One mechanism that VEGF may utilize to induce angiogenesis is that it may post-transcriptionally activate PI3 kinase and platelet and endothelial cell adhesion molecule-1 (PECAM-1) expression. PECAM-1 is a crucial protein in human endothelial cells [38]. In addition, in human colonic cancer endothelial cells, sphingosine-1-phosphate (S1P), an important GSL involved in cell growth and proliferation, was shown to induce angiogenesis [13]. The same result was also achieved through the incubation of exogenous LacCer, irrespective of the presence of various S1P inhibitors. This suggests that LacCer-mediated angiogenesis is independent of S1P and VEGF.

Thus, it was later discovered that β-1,4-GalT-V is the major LacCer synthase found in human tumor endothelial cells, and its transcript increases 4.5-fold in these cells [38]. VEGF was found to stimulate LacCer synthesis through β-1,4-GalT-V in a time-dependent manner, and adding a β-1,4-GalT-V inhibitor (D-PDMP) mitigated VEGF-induced LacCer biosynthesis as well as VEGF-induced angiogenesis in endothelial cells [9]. Within minutes of VEGF binding to its receptor on the surface of human endothelial cells, β-1,4-GalT-V can be activated [9]. This pathway of VEGF-induced angiogenesis through LacCer is an independent pathway from other molecules involved in angiogenesis, like S1P and S1K [38]. Interfering with the angiogenesis pathway may be a promising therapeutic strategy for cancer treatment.

### 2.5. Effects of β-1,4-GalT-V Gene Deletion and Overexpression

Studies show that angiogenesis is critical in embryonic development. β-1,4-GalT-V has been identified as a major player in angiogenesis and is required for endothelial function, so there have been studies examining β-1,4-GalT-V’s role in embryonal development [39]. In one study, mouse blastocysts were treated with tunicamycin, which inhibits N-glycan biosynthesis. This caused developmental arrest in these mice, which suggested that development is reliant on these glycans. Based on various glycosyltransferase-knockout mice, β-1,4-GalT-V was indicated to be involved in the galactosylation of N-glycans in vivo. Moreover, β-1,4-GalT-V-knockout mice are eliminated at early post-implantation stages, whereas β-1,4-GalT-I-knockout mice survive until after birth. Therefore, products synthesized by β-1,4-GalT-V are hypothesized to be important for placental formation and/or hematopoietic development, which are essential for embryonic development [40].

It was shown that in human umbilical vein endothelial cells, ablating β-1,4-GalT-V through siRNA-silencing techniques mitigates VEGF-induced PECAM-1 protein expression and, ultimately, angiogenesis. This is a crucial finding in demonstrating that β-1,4-GalT-V is essential to angiogenesis [41]. Second, in a mouse model of glioblastoma, the use of shRNA for β-1,4-GalT-V markedly reduced brain tumor volume [26]. Sato reported that β-1,4-GalT-V gene deletion in cancer cells is reduced. Most importantly, they also showed that a knockout mutation may be lethal in one highly inbred mouse strain but not in another because compensatory pathways exist in the latter. β-1,4-GalT V-knockout mice are embryonically lethal, suggesting the importance of the glycans synthesized by β-1,4-GalT-V for embryonic development [42].

In another study, a double knock-out of β-1,4-GalT-V and β-1,4-GalT-VI genes in the central nervous system of mice significantly reduced embryogenesis and was lethal, as only 0.9% of the total pups survived 3 weeks [42]. It was hypothesized that these double-knockout mice developed normally during embryogenesis and perinatal stages but had motor defects and exhibited growth retardation. One major reason for this is the inability to produce LacCer, which resulted in severe depletion in the level of its higher homologs, e.g., gangliosides, thus limiting interactions with laminin and immature neurons. On the other hand, β-1,4-GalT-VI gene knockout in mice did not impair pregnancy, embryonal growth, health, and fertility. The expression levels of β-1,4-GalT-VI also appeared to be uninfected in β-1,4-GalT-V conditional gene knockout, meaning that these two proteins work in different pathways, and β-1,4-GalT-VI function is unable to compensate for β-1,4-GalT-V knockout. These studies demonstrated that overall, the β-1,4-GalT-V gene (enzyme/protein) is central to mammalian health and embryonic survival. Therefore, dysregulation of the β-1,4-GalT-V gene can cause various diseases, particularly cancer, cardio-vascular disease, and other developmental disorders.

### 2.6. Oxidative Stress, Sheer Stress, and β-1,4-GalT-V

Although it was shown that human arterial endothelial cells overexpress ICAM-1 upon exposure to fluid sheer stress, the earlier steps in this pathway were not known. In a study wherein these cells were subject to hemodynamic/fluid sheer stress (20 dynes/cm^2^), there was a time-dependent increase in the activity of β-1,4-GalT-V and LacCer generation. This was followed by the activation of NADP(H) oxidase; superoxide/O_2_^−^ generation; Ras-GDP loading; and JNK, ERK, NF-kB, c-jun, c-Fos, and AP-I activation—this led to ICAM-1 expression [43]. Since ischemia-reperfusion injury involves the mitochondria, an early rise in ceramide levels can accompany an increase in the level of LacCer by the activation of β-1,4-GalT-V. In a study, it was shown that Rac1gene inhibition blocks hypoxia/reoxygenation-induced lipid peroxidation in these cells, suggesting that ischemia-reperfusion injury may well involve β-1,4-GalT-V [43].

### 2.7. Environment/Lifestyle Habits and β-1,4-GalT-V

Life-style habits such as regularly enjoying a high-fat and high-cholesterol diet (Western diet) easily available from fast food chains can contribute to dyslipidemia and inflammatory oxidative stress, contributing to the greying of hair, hair loss, and skin lesions in men. Thus, nearly 40 percent of adult, middle-aged males become bald. We observed that by feeding a Western diet to the apolipoprotein E mutant (ApoE^−/−^), a transgenic mouse model of atherosclerosis, we can reproduce the progressive greying and hair loss observed in men accompanied with wounding of skin [44]. We also observed that inflammation in the skin in these mice was caused by a pro-inflammatory cytokine TNF-α. Additionally, TNF-α induced the secretion of a stimulatory factor (TSG), known to have a hyaluronan binding domain associated with cell migration and inflammation. Neutrophils are blood cells that play a critical role in innate immunity and inflammation. Thus, neutrophil infiltration into a tissue may well serve as a hallmark of inflammation.

We observed neutrophil infiltration into the dermal area in the skin in these mice fed a Western diet. As neutrophils carry the bulk of LacCer, a neutrophil burst may release LacCer into the inflamed tissue to add to the oxidative stress environment and inflammation. Moreover, LacCer can markedly induce the expression of CD11b on the surface of neutrophils and monocytes on one hand. And on the other hand, LacCer can also increase the expression of cell adhesion proteins, e.g., intercellular cell adhesion molecule (ICAM-1) on the surface of human endothelial cells and platelet cell adhesion molecule, PECAM-1, a cell junctional protein implicated in the transmigration of neutrophils and monocytes. This occurs as ICAM-1 and PECAM-1 serve as receptors to CD11b in neutrophils [45]. This enables activated endothelial cells to overexpress these cell adhesion molecules to capture neutrophils and monocytes.

Most importantly, we observed that feeding a Western diet raised the mass of β-1,4-GalT-V protein but not β-1,4-GalT-V gene expression in skin tissue. This, in turn, had a domino effect by utilizing and consequently decreasing the skin level of ceramides (the major pro-hydration lipid in the skin) and glucosylceramide but raising the mass of LacCer. Such LacCer activated phospholipase A-2, an enzyme that cleaves a phospholipid called phosphatidylcholine to generate arachidonic acid, and downstream eicosanoids and prostaglandins, powerful inflammatory agonists [46]. Conversely, treatment with BPD dose-dependently decreased β-1,4-GalT-V mass and LacCer mass, thus restoring ceramide levels and improving skin health. Infection due to pneumocystis bacteria is a common occurrence in mouse colonies. Interestingly, these ApoE^−/−^ mouse colonies fed BPD did not have any infection or any other infections. Thus, immunity was not compromised in our ApoE^−/−^ mice, but treatment with BPD clearly mitigated inflammation. And this tenet was examined further in another study below. In sum, a Western diet increases β-1,4-GalT-V activity/mass and raises the skin level of LacCer, which induces neutrophil infiltration into the skin dermis, causing inflammation accompanied by significant hair discoloration/loss and skin wounding. Conversely, treatment with BPD, which blocks β-1,4-GalT-V activity and mass in the skin, completely reversed skin pathology induced by feeding a Western diet [44].

## 3. Inflammation and β-1,4-GalT-V

Chronic inflammation has been found to regulate many diseases, including cardiovascular diseases, diabetes, Alzheimer’s, and cancer. Inflammation is the body’s response to tissue damage from physical or ischemic injury, infection, toxin exposure, and other trauma [47]. In the recent literature, β-1,4-GalT-V has been implicated in different inflammation pathways, which is also an important connection to β-1,4-GalT-V’s role in cancer.

### 3.1. Inflammatory Molecules and β-1,4-GalT-V

Tumor necrosis factor-alpha (TNF-α) is by far the most investigated among all pro-inflammatory molecules. TNF-α is a cytokine produced by cells upon exposure to lipopolysaccharide and/or bacterial infection as an immune-defense measure. Further, TNF-α binds to its receptors to induce programmed cell death/apoptosis. TNF-α can also activate the phosphokinase signaling pathways and cause cell proliferation. For example, in rat primary astrocytes, TNF-α increased the activity of LacCer synthase and increased cell proliferation via recruiting PI3 kinase Ras and MAPK. And this was mitigated upon pre-treating the cells with D-PDMP. Such studies were duplicated in a mouse model of spinal cord injury [48,49]. Cultured astrocytes that do not have TNF-α and TNFR1 treatment can produce both upon stimulation by lipopolysaccharides. This was accompanied by increased expression of β-1,4-GalT-I and -V mRNAs [50]. More recent studies have shown that the cortex, hippocampus region, and microglia cells express β-1,4-GalT-V and play an important role in “neuro-inflammation” by way of proliferation and hyper-activation [51].

**Figure 5:**
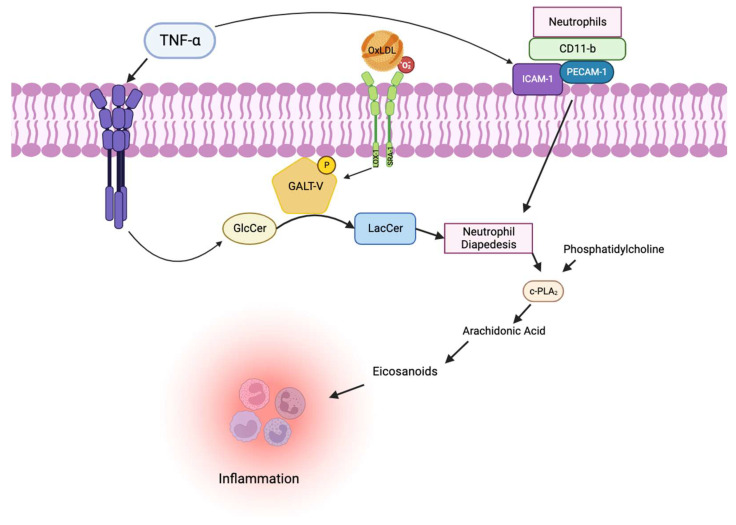
Inflammation pathways involving β-1,4-GalT-V. This figure shows the biological pathway by which both ox-LDL and TNF-α activate β-1,4-GalT-V. Phosphorylated β-1,4-GalT-V produces LacCer, initiating signaling for increased neutrophil diapedesis. Inside endothelial cells, LacCer activates c-PLA2, leading to arachidonic acid production. Arachidonic acid is a precursor for inflammatory eicosanoids, amplifying inflammation. TNF-α also active PECAM-1 and ICAM-1, aiding the inflammation pathway [52].

The binding of TNF-α to cultured human umbilical vein endothelial cells not only increases the activity of β-1,4-GalT-V but also increases the expression of multiple cell adhesion molecules, e.g., intercellular cell adhesion molecule (ICAM-1), PECAM-1, endothelial cell adhesion molecule (ELAM-1), vascular cell adhesion molecule (VCAM-1), and P-selectin. Some of these cell adhesion molecules, e.g., ICAM-1/PECAM-1 expressed on the cell surface in endothelial cells, serve as receptors to CD11/CD18, also expressed on the cell surface in monocytes and neutrophils. After the adhesion of monocytes to endothelial cells, PECAM-1 and ICAM-1 further facilitate the entry of monocytes into the endothelium, wherein it can release its cargo, including LacCer that can independently increase angiogenesis. On the other hand, LacCer can activate cytosolic phospholipase A2 (cPLA2) to cleave phosphatidylcholine and release arachidonic acid, a precursor to prostaglandins, and exacerbate inflammation (Figure 5).

The observation that inflammatory cells are found abundantly in tumor tissue suggests that cancer originates in sites of chronic inflammation [53,54]. Thus, tumor growth and response to treatment are regulated by inflammation. Acute inflammation increases dendritic cell maturation as well as antigen presentation, thus resulting in an anti-tumor response to a treatment. Additionally, the Toll-like receptors (TLR), MAPK, and JAK-STAT pathways are all implicated in cancer and inflammation. Moreover, pro-inflammatory cytokines, e.g., IL-1, IL-6, IFN gamma, C-C motif chemo-like ligands (CCLs), prostaglandins, VEGF, TGF-B, and inflammasome, all partake in cancer and inflammation. In sum, inflammation plays a significant role in the initiation, progression, and treatment of cancer [55,56].

### 3.2. Cell Adhesion and β-1,4-GalT-V

As β-1,4-GalT-V has a role in many physiological pathways, one related phenotype is cell adhesion, which is important in atherosclerosis and other diseases. Another aspect of the pathogenesis of atherosclerotic lesion formations, along with inflammation, is cell adhesion. TNF-α has been well documented in inducing cell adhesion molecule expression by activating an oxidant-sensitive signal transduction pathway through β-1,4-GalT-V [9]. These cell adhesion molecules include intercellular cell adhesion molecule-1 (ICAM-1), vascular cell adhesion molecule-1 (VCAM-1), and platelet cell adhesion molecule (PECAM-1), which all go on to regulate cell proliferation and angiogenesis [38].

More specifically, TNF-α increases LacCer synthesis in endothelial cells in a time- and concentration-dependent manner through stimulating β-1,4-GalT-V. Cells incubated with D-PDMP inhibited TNF-α induced ICAM-1 expression in a concentration-dependent manner. However, when exogenous LacCer was introduced, this inhibition was reversed, suggesting that β-1,4-GalT-V and LacCer are crucial in cell adhesion [38]. In addition, when PDMP was introduced in cultured media (CM) astrocytes, which are critical players in CNS immune response, ICAM-1 and VCAM-1 mRNA and protein levels were decreased. When β-1,4-GalT-V was directly inhibited, the activation of TF in astrocytes was also inhibited. Astrocytes’ interaction with endothelial cells in the formation of the blood–brain barrier is crucial to the neuroinflammation response, and β-1,4-GalT-V is a key player in these cell adhesion and inflammation processes [57].

### 3.3. Lupus Erythematosus and β-1,4-GalT-V

Systemic lupus erythematosus is an autoimmune disease due to inflammation in several organs, e.g., heart, skin, kidney, and brain. Studies show that genetics, environmental risk factors, and hormones play a critical role in this disease and may lead to atherosclerosis. And death in late-stage SLE is due to myocardial infarction and atherosclerosis. Yet the increased risk for SLE cannot be explained using Framingham risk factors, e.g., cholesterol, blood pressure, high-density lipoprotein, diabetes, cigarette smoking, and cardiac hypertrophy. On the other hand, an increased level of lipoprotein a (Lp(a)) has been associated with SLE [52]. Lp(a) is a small protein attached to apolipoprotein B (ApoB) in an LDL molecule and enhances thrombogenesis. Interestingly, it also serves as a carrier of pro-inflammatory molecules, including oxidized phospholipids, which we had shown to activate β-1,4-GalT-V (see above). These observations prompted us to carry out a case–control study in serum samples from women with SLE and healthy controls. These studies revealed that SLE patients have increased levels of β-1,4-GalT-V, Lp(a), and oxidized phospholipids compared to healthy controls (*p* < 0.0001).

### 3.4. Phagocytosis, COPD, and β-1,4-GalT-V

Phagocytosis is a form of autophagy wherein a cell membrane invaginates to capture bacteria, apoptotic cells, and foreign substances and internalizes into the cytoplasmic bodies called a phagosome or endosome. Thus, phagocytosis is essential to maintain homeostasis in tissues. Autophagy can be tracked using a biomarker, e.g., p62, a protein, and/or LC3B and ubiquitinated protein aggregates. Studies show that cigarette smoke can raise the tissue level of LacCer as well as p62 in an experimental model. Additionally, LacCer accumulation has been associated with increasing severity of emphysema in chronic obstructive pulmonary disease (COPD) and lung injury. Thus, increased levels of LacCer may serve as an important pathogenic biomarker in COPD-emphysema pathogenesis.

This study used human bronchial, epithelial cells (BEAS2B), and macrophage (Raw264.7) cells exposed to cigarette smoke extract (CSE), as well as murine lung injury and emphysema models (C57BL/6) mice exposed to acute sub-chronic CS and Pseudomonas aeruginosa lipopolysaccharide ((Pa)-LPS). Treatment with CSE significantly increased the cellular level of LacCer in bronchial epithelial cells and macrophages. Conversely, treatment with D-PDMP reduced the number of CSE-induced p62-positive cells compared to the control. Additionally, CSE treatment induced co-localization of LC3-GFP and ubiquitinated protein RFP and apoptosis. Conversely, treatment with D-PDMP inhibited LC3-Ub accumulation, thus confirming the critical role of LacCer as well as β-1,4-GalT-V in CSE-induced autophagy response and apoptosis. These findings were further verified using Pa-LPS-induced emphysema and lung inflammation in C57BL-6 mice.

In sum, this study demonstrated that LacCer accumulation in the lung due to CSE and/or Pa-LPS exposure in mice is a hallmark in inflammatory/apoptotic response contributing to aberrant autophagy. These studies imply that by using D-PDMP to block β-1,4-GalT-V activity, this enzyme is the target for CSE and Pa-LPS-induced dysregulation of autophagy that underlies the pathology–biochemical mechanism of COPD emphysema pathogenesis [25]. Moreover, treatment with D-PDMP mitigated infection due to Pa-LPS. Collectively, these observations made using ApoE^−/−^ mice fed a high-fat and high-cholesterol diet and C57Bl6 mice infected with Pa-LPS and treated with BPD or D-PDMP suggest that targeting β-1,4-GalT-V is a novel approach to mitigate inflammation without compromising immunity.

## 4. β-1,4-GalT-V and Cancer

### 4.1. Stem Cells and β-1,4-GalT-V

Aside from the galactosylation of glucosylceramide to form LacCer, β-1,4-GalT-V also galactosylates the β 1to β 6 branch arm of the highly branched N-glycan residues in glycoproteins. Since the malignant transformation of such N-glycan accompanies malignant transformation, it suggests that β-1,4-GalT-V has a pivotal position in stem cell cancers. This tenet is substantiated by reports on breast, prostate, hematopoietic, pancreatic, neck, colorectal, and brain cancers [58], wherein β-1,4-GalT-V is overexpressed compared to healthy subjects. In addition, glioblastomas are known to express 10-fold more of β-1,4-GalT-V than other cancers. This is because of the self-renewal of glioma-initiating stem cells. Conversely, downregulation of β-1,4-GalT-V using corresponding shRNA dose-dependently reduced β-1-4 GalT-V mRNA and protein in a mouse xenograft model of glioblastoma. This was accompanied by reduced cell proliferation in mice bearing gliomas [26]. As glioma-initiating stem cells are characterized by neural stem cell markers, e.g., nestin and CD133, downregulation of β-1,4-GalT-V was also accompanied by reduced mRNA expression of these neural stem cell markers, suggesting that CD133 and nestin may well have β-1-4 galactosylated epitope. And indeed, Western immunoblot assays of xenograft tumor samples from β-1-4 GalT-V shRNA treated mice revealed a decrease in the mass of CD-133 and nestin. In sum, this study revealed that stem cell biomarkers, e.g., CD133 and nestin, may well have a β-1,4-GalT epitope. And that malignant transformation of stem cells may involve galactosylation, resulting in overexpression of these proteins and consequent increase in phenotypes, e.g., proliferation, migration, angiogenesis, and tumor malignancy.

These studies opened the possibility that there may be other phenotypes wherein the β-1,4-GalT epitope may well regulate tumorgenicity. A case in point was Notch1, which was implicated in a demonstration that N-glycosylation regulates cell behavior through regulating membrane protein signaling. Notch1 is a glycoprotein, an N-glycan signaling pathway known to monitor cell differentiation, proliferation, and apoptosis. Studies show that N-glycation of Notch1 can regulate Notch-1 stability. To examine the role of β-1,4-GalT-V in Notch1 galactosylation, β-1,4-GalT-V expression was depleted using shRNA in a primary culture of human brain tumor cells (T698968). Depletion of β-1,4-GalT-V blocked β-1,4-galactosylation of Notch1 associated with a decrease in the migration of Notch1 to the cell surface. It binds to Galectin-3, as Notch1 binding to Galectin is also found to be galactosylation-dependent [59]. Thus, reduced galactosylation of Notch1 resulted in decreased degradation. Since Notch1 antibody immunoprecipitated β-1,4-GalT-V suggests that Notch-1 may be a substrate for β-1,4-GalT-V-mediated galactosylation (Figure 4).

One study also showed that β-1,4-GalT-V depletion blocked the trans-differentiation of glioma stem cells into endothelial cells and possibly angiogenesis [60]. Inactivation of this pathway with y-secretase or Notch1 knockdown leads to inhibition of glioma stem-like cells into endothelial cells. Recently, it was found that glioma stem-like cells transdifferentiate into tumor vascular endothelial cells, which was indicated as a new mechanism for VEGF-independent angiogenesis in malignant glioblastoma. N-glycans play a large role in regulating the activation of Notch signaling by manipulating the Notch receptor–ligand interaction. A study explores the role of β-1,4-GalT-V in gliomas (its trans-differentiation of glioma stem-like cells into endothelial cells) and the mechanism of Notch to develop better therapeutic drugs for this aggressive human cancer. It is important because it was shown, for the first time, that β-1,4-GalT-V can regulate trans-differentiation of glioma stem-like cells into endothelial cells, and depleting β-1,4-GalT-V was a key step in improving the survival of mice with glioblastoma tumors. N-glycan in tumor angiogenesis has been accumulating evidence as playing a critical role in tumor angiogenesis. β-1,4-GalT-V was also found to regulate Notch1 signaling in the trans-differentiation process of glioma stem-like cells into vascular endothelial cells (Figure 4); by decreasing the expression of β-1,4-GalT-V, Notch1 cleavage was reduced. Although the mechanism is currently unknown, the connection between β-1,4-GalT-V and Notch1 was discovered in this study, which is crucial to tumor angiogenesis, leading to possible cancer therapies [60].

### 4.2. Cell Migration and β-1,4-GalT-V

Members of the Frizzled family of integral membrane proteins are implicated in many developmental events, including specifying cell fate, orienting cell/planar polarity, and directing cell migration. Members of the Frizzled family function as cell surface receptors for secreted Wnt proteins. β-1,4-GalT-V glycosylates Frizzled1 (Figure 4), which plays a crucial role in promoting cell migration. Secreted Frizzled-related protein 1 (SFRP1) is a member of the SFRP family that modulates the Wnt signal transduction pathway. Downregulation of SFRP1 expression has been observed in CRC. Studies have shown that SFRP1 also suppresses cell proliferation, migration, and invasion and promotes apoptosis in CRC cells. Changes in the N-glycan structure of glycoproteins in glioma cells affect cell migration and tumor malignancy. GnT-V activates EGF-mediated signaling and, in part, promotes cell migration through the modification of N-glycans on receptor protein tyrosine phosphatase kappa (RPTPκ). Studies have shown that membrane-proximal N-glycosylation on integrin β1 positively regulates cell migration by promoting β1 activation. This suggests a novel regulatory mechanism wherein N-glycosylation near the cell membrane on β1 may serve as a platform that facilitates its complex formation on the cell membrane, thereby affecting integrin-mediated functions [61].

### 4.3. Upregulation of β-1,4-GalT-V in Cell Proliferation and Tumor Growth

In addition to aberrant glycosylation, it has been discovered that β-1,4-GalT-V also participates in cellular processes as an oncogenic signal transducer. This was demonstrated in CRC, where β-1,4-GalT-V exhibits increased expression levels (approximately 6.5-fold) as compared to normal tissues. There is also an increase in its enzymatic activity and its product LacCer in CRC [13]. As a matter of fact, while studies in many human cancer cell lines have shown β-1,4-GalT transferases to have little change in enzymatic activity upon malignant transformation, the same studies show β-1,4-GalT-V to have a 2-3-fold increase in cancer cells such as NIG 3T3 and MTAg. Furthermore, similar studies found that human cancer cells showed up to 5 times more levels of increase of β-1,4-GalT-V. In a human colorectal cancer cell line (HCT-116), the gene/protein expression of β-1,4-GalT-V, LacCer mass, and cell proliferation has a cell density-dependent increase, which was concomitantly mitigated by treatment with D-PDMP. In addition, another study revealed that the mRNA levels of β-1,4GalT-V were specifically increased in human colorectal cancer, contributing to tumor growth and metastasis. There was also an upregulation of both β-1,4-GalT-V activity and mass, as well as a most noticeable increase in the level of LacCer shown in human colorectal cancer tissues [13]. Thus, an overexpression of β-1,4-GalT-V was shown to increase tumor growth, while suppression by gene ablation diminished tumor growth. Furthermore, β-1,4-GalT-V upregulation could also lead to the increased activity of several other CRC gene biomarkers, which is crucial to the diagnosis and treatment of CRC. This could also be generalized to other forms of cancer as well [16].

β-1,4-GalT-V was also revealed to be highly enriched in vascular tissues—especially in single layers of endothelial cells, which take up most of the surface of the blood capillaries and are directly exposed to circulating blood. Large amounts of β-1,4-GalT-V are also associated with the cytoplasm in human colorectal cancer cells. When a non-coding DNA strand of β-1,4-GalT-V was introduced into cancer cells in an animal model, the tumor development was suppressed. Patients with inflammatory bowel disease (IBD) who have a greater risk of developing CRC have an elevated count of β-1,4-GalT-V. It can become an important biomarker for patients [9]. As shown in previous studies, VEGF was shown to stimulate β-1,4-GalT-V and to generate LacCer, eventually leading to angiogenesis using an “oxygen sensitive signaling” pathway. Tumors need VEGF to facilitate angiogenesis in many tumor types. One study connected the findings of recent studies to tumor growth as well as cell proliferation to investigate whether inhibiting glycosphingolipid synthesis would mitigate these phenotypes. It was shown that inhibiting LacCer synthase activity and reducing LacCer may reduce renal cancer in mice. Moreover, from these results, it was shown that β-1,4-GalT-V activity leading to LacCer production is central to signaling events involved in angiogenesis and cell proliferation, which led to decreased renal cancer tumors.

## 5. Genetic Basis of Cancer Pathways Using the OMIM Database

Online Mendelian Inheritance in Man (OMIM) is a comprehensive database of human genes and genetic disorders used to provide information about the genetic basis of inherited diseases and traits, as well as information about the molecular pathways and biological processes that underlie human physiology. It contains detailed descriptions of over 15,000 genetic disorders, including information about the clinical features, inheritance patterns, molecular basis of the condition, and chromosomal location of genes involved. The complete list of cancer genes interacting with β-1,4-GalT-V and its gene family, as generated by OMIM, can be found here: sorted omim data unfiltered. Taking all this information into account, we specifically looked at genes that interact with and are targeted by β-1,4-GalT-V and its families, especially in cancer pathways. This is listed in Table 1.

The interconnectedness of oncogenes with β-1,4-GalT-V is evidently seen in the pathways they share and similar processes that regulate them. Oncogene regulation is intertwined with metabolic factors, making their study vital for a comprehensive understanding of cellular dynamics, where processes like glycolysis and metabolism are quintessential to maintaining cellular homeostasis. Table 2 describes such interactions in more detail.

The vitality of glycogenes is seen in pathways beyond just cancer and heart disease. From skin diseases to eye ailments, glycogenes have a quintessential role in regulating signaling in a variety of pathways. Targeting these pathways will allow us to treat these maladies in a timely fashion and with greater efficacy. Table 3 further describes such diseases of interest and provides context for the role of glycogenes in treating or diagnosing such ailments.

## 6. Cancer Therapies Targeting β-1,4-GalT-V

Common cancer treatment modalities, such as γ-irradiation and chemotherapeutic agents, induce cell death via the generation of ceramide. As the central hub of sphingolipid metabolism, β-1,4-GalT-V, its homologs, and different agents like ceramide will be instrumental in innovative cancer treatments by working as a diagnostic biomarker and target.

### 6.1. Drug Therapies

#### 6.1.1. Chemotherapy

Ze-Jin Ma and Li Liu mentioned broad-spectrum chemotherapy: a novel approach using β-galactosidase activated pro-drugs [69]. It has been expected that administering agents to target ceramide metabolism in combination with chemotherapy will improve response rates, especially in those patients with metastatic disease. Tilly and colleagues, using an inhibitor of ceramide-induced cell death, showed that cell destruction by chemotherapy was preventable if ceramide metabolism was manipulated [70]. Several key ceramide-metabolizing enzymes have been shown to be upregulated in chemotherapy-resistant acute myeloid leukemia (AML) cells compared to chemotherapy-naïve counterparts. Fisher-Wellman et al. also studied the effects of blocking ceramide clearance in drug-resistant AML by employing simultaneous inhibition of ceramide hydrolysis and ceramide glycosylation [71]. They demonstrated that this dual blockade produced multi-fold elevations in intracellular ceramide levels, enhanced Caspase activation, and elicited cell death. Numerous studies now clearly demonstrate that co-administration of agents that target ceramide metabolism enhances the cytotoxic impact of chemotherapy. For example, vinblastine toxicity is intensified by SDZ PSC 833 in P-glycoprotein-poor cells, and both drugs promote ceramide formation at low doses. Doxorubicin and tamoxifen synergize to increase ceramide formation, complementing cytotoxicity. Senchenkov (2001) also showed how the cytotoxic effect of chemotherapy is decreased when ceramide generation is impaired but is increased when ceramide degradation is blocked [72]. Chemotherapeutic drugs and radiotherapy serve to sensitize cancer cells and induce apoptosis by restoring ceramide levels [73].

#### 6.1.2. Immunotherapies

The goal of immunotherapy is to strengthen a patient’s immune system to attack tumors more efficiently. It has quickly risen to become a pillar in cancer treatment since it has been an effective way to shrink or even eradicate tumors in many patients [74]. Aberrant glycosylation in cancer may occur in both glycoproteins and glycosphingolipids. Among the best characterized tumor-associated carbohydrate antigens (TACAs), we found truncated O-glycans (Thomsen-nouveau, Tn; Thomsen–Friedenreich, TF; and sialyl-Tn, STn), gangliosides (GD2, GD3, GM2, GM3, fucosyl-GM1), Globo-serie glycans (Globo-H, SSEA-3, SSEA-4), Lewis’s antigens, and Poly sialic acid. For more than twenty years, these TACAs have demonstrated potential usefulness in strategies for cancer immunotherapy. Most TACAs are poorly immunogenic, inducing T cells’ independent immune responses; they must be conjugated to carrier proteins or be chemically modified to induce an effective antitumor immune response. The main glycosphingolipids classified as TACAs are Globo H, the stage-specific embryonic antigens-3 and -4 (SSEA-3 and SSEA-4), and the GSL containing sialic acid, such as the gangliosides GD2, GD3, GM2, fucosyl GM1, and Neu5GcGM3. These GSLs can affect cancer development by controlling cell adhesion, motility, and growth; the epithelial–mesenchymal transition; metastatic development; as well as drug resistance. Globo-H ceramide (GHCer) is overexpressed in several cancers, including breast, gastric, lung, ovarian, endometrial, pancreatic, and prostate cancers. GHCer could be transferred from cancer cells into non-tumor cells located in the tumor microenvironment, such as endothelial cells, promoting angiogenesis, as well as into T cells, inhibiting IL-2, interferon-γ, and IL-4 secretion, promoting immunosuppression. The specific expression of GHCer in tumor cells, as well as its role in promoting tumor progression, makes this antigen an attractive target for anticancer immunotherapies. Invariant natural killer T cells (iNKT) are T cells that can recognize glycolipid antigens, such as α-galactosylceramide (αGalCer). In vivo activation of iNKT cells with αGalCer results in robust cytokine production [75]. Parekh et al. found that αGalCer-activated iNKT cells rapidly upregulated the expression of PD-1. PD-1: PD-L blockade prevents energy induction and enhances the anti-tumor activities of αGalCer-activated iNKT cells [76].

### 6.2. CRISPR/Cas9

CRISPR-Cas9 is a gene editing technology that enables scientists to modify DNA sequences in living organisms selectively. CRISPR-Cas9 has two main components: the Cas9 protein and a guide RNA (gRNA). The gRNA is designed to recognize and bind to a specific target DNA sequence that will be edited, while the Cas9 protein acts as a molecular scissor that cuts the DNA at the targeted site. After the DNA is cut, homology-directed repair can be used to delete, replace, or add new DNA sequences at the cut site. This allows for precise genetic changes that can be used for studying gene function, developing new therapies for genetic diseases, and potentially editing human embryos [77]. Glycosylation is essential for the interaction of PD-1 and PD-L1. By using the genome-wide loss-of-function screening strategy based on the CRISPR-Cas9 system, researchers identified genes involved in the core fucosylation pathway as positive regulators of cell-surface PD-1 expression. N49 and N74, of the core fucosylated N-glycans for PD-1, are determined as the significant locations, inhibition of fucosyltransferase 8 (Fut8), a key fucosyltransferase, reduced PD-1 expression and enhanced T-cell activation.

### 6.3. Antibody–Drug Conjugate (ADC)

A recent treatment of cancer called antibody–drug conjugate is a new method of therapy that uses monoclonal antibodies that are chemically attached to a cytotoxic drug. Using antibodies to target tumor surface markers allows highly specific targeting ability to effectively eliminate cancer cells. This technique is still in the testing stages, and currently, there are 14 ADCs that have received market approval worldwide. There are over 100 ADCs currently being studied, and this therapy could be highly effective in the future.

### 6.4. Cancer Immune Checkpoints

Glycosylation of immune checkpoint molecules has become a promising target for tumor immunotherapy. New drugs targeting glycosylation have been developed with in vivo and in vitro trials underway, and some drugs have already been initiated in clinical trials. Most immune checkpoint molecules can be modified by glycosylation, with the B7-CD28 superfamily being particularly notable. It belongs to the co-stimulatory molecule families and plays a vital role in tumor immunotherapy. These co-stimulatory pathways can either provide agonistic signals to enhance and maintain T-cell immune responses or provide inhibitory signals to suppress T-cell immunity responses. Ipilimumab is an anti-CTLA-4 monoclonal antibody used in cancer immunotherapy. Like PD-1 and PD-L1, CTLA-4 can also undergo glycosylation, which can impact its interaction with B7 ligands. Ceramide is a regulator of the taxane-mediated spindle assembly checkpoint and taxane-induced cell death [75]. Paclitaxel-treated MCF-7 cells activated acid sphingomyelinase (ASMase), leading to ceramide production that, in turn, results in a reduction in cell motility [76].

### 6.5. Car T-Cell Therapy

Chimeric antigen receptor (CAR) T-cell therapy has created a paradigm shift in the treatment of hematologic malignancies but has not been as effective toward solid tumors. For such tumors, the primary obstacles facing CAR T cells are the scarcity of tumor-specific antigens and the hostile and complex tumor microenvironment. Glycosylation, the process by which sugars are post-translationally added to proteins or lipids, is profoundly dysregulated in cancer. Abnormally glycosylated glycoproteins expressed on cancer cells offer unique targets for CAR T therapy as they are specific to tumor cells. Tumor stromal cells also express abnormal glycoproteins and thus also have the potential to be targeted by glycan-binding CAR T cells. CAR T cells combine the specificity of an antibody with the cytolytic capacity of T cells in an MHC-independent manner. These therapies have proved successful for hematological malignancies, but in solid tumors, multiple challenges need to be overcome, including the identification of new tumor-associated antigens, the limited infiltration of CAR T cells to tumor sites, and the immunosuppressive effect of the tumor microenvironment. At the beginning of the century, Rossig et al. published one of the first CAR-targeting GD2, demonstrating in vitro that first-generation CAR T cells derived from GD2a antibodies recognized and lysed GD2-positive cancer cells in an antigen-specific manner [78]. The first clinical trial used Epstein–Barr Virus (EBV)-specific T cells, engineered with first-generation CAR-targeting GD2, and recruited 11 patients with neuroblastoma. Further, reducing N-linked glycosylation of PD-1 may decrease PD-1 expression and relieve its inhibitory effects on CAR T cells [79]. N-glycans provide resistance to CAR T-cell therapy, and their inhibition improves CAR efficacy. Reduction of extracellular N-glycans could improve CAR T-cell therapies in solid malignancies [80].

## 7. Perspectives

From the above discussion, we have only addressed the “tip of iceberg” about the role of β-1,4-GalT-V in mammalian health and diseases. Since β-1,4-GalT-V has now been implicated in inflammatory pathways and various critical phenotypes regulating embryogenesis, growth, aging, disease onset, and death, it rationalizes further research to understand genes that regulate this enzyme and post-transcriptional modifications that affect function and localization. What factors regulate/determine β-1,4-GalT-V enrichment in various types of cancer? Does β-1,4-GalT-V glycosylate a host of proteins implicated in the regulation of various phenotypes? The role of this enzyme in drug resistance must be answered in detail. There is an urgent need to have highly specific inhibitors for β-1,4-GalT-V and to develop drug delivery systems and various recent approaches listed above for therapy to mitigate solid tumors and multiple diseases in animal models and eventually in humans. In view of the possibility of multiple and spontaneous mutations that can occur within a tumor, how to tailor “Precision Medicine” in this context remains an enormous challenge to tackle mortality and morbidity in cancer patients.

## 8. Conclusions

Since the late 1970s, Dr. Chatterjee’s lab and many others have been discovering more about β-1,4-GalT-V and its role in disease development. Our contribution to this field of research is the following:

1. We demonstrated that various physiologically relevant molecules, Western diet, sheer stress, and cigarette smoke all converge upon β-1,4-GalT-V to generate LacCer.
2. LacCer is a bonafide bioactive signaling molecule that can increase angiogenesis, proliferation, migration, phagocytosis, and apoptosis—all the critical phenotypes in health and disease. Additionally, and most importantly, LacCer can activate the inflammatory pathway by way of activating cytosolic phospholipase A2 to generate arachidonic acid, eicosanoids, and prostaglandins, as well as neutrophil infiltration into tissues.
3. The use of inhibitors of β-1,4-GalT-V and siRNA and shRNA can mitigate nearly all these phenotypes and improve cardiovascular health glucose homeostasis in type II diabetic mice and mouse models of CRC.

With new technologies like gene sequencing and the availability of mouse models of human disease, the field has rapidly grown in discoveries related to β-1,4-GalT-V and major human disease pathways, like cancer, cardiovascular disease, and a whole host of inflammation-centric diseases.

4. We propose that β-1,4-GalT-V protein is a central gateway, a uniter wherein multiple risk factors and growth factors converge, and that the LacCer generated consequently activates numerous signaling pathways to induce inflammation to exacerbate diverse diseases, e.g., cancer, cardiovascular disease, and inflammatory diseases.
5. Using genetic information provided by the OMIM database is another way to strengthen our information repository and further investigate β-1,4-GalT-V’s pivotal role. Moreover, since inflammation nearly always accompanies cardiovascular diseases and cancer progression, a better understanding of β-1,4-GalT-V is warranted to develop novel approaches to mitigate these diseases as well as for their early diagnosis.

## Figures and Tables

**Table 1:** Linkage between glycogenes and signal transduction pathways.

Glycogene	Role Played by Glycogene in Pathway
β-4-GalNT-I	Its expression may play a role in tumor microenvironment and tumor immunity regulation in multiple types of cancer. It enhances proliferation and suppresses apoptosis of oral squamous cell carcinoma cells through JNK and p38 signaling pathways.
β-3-GalNT-II	It promoted tumor growth by enhancing macrophage recruitment in vivo. In breast cancer cells, it is N-glycosylated on both Asn-116 and Asn-174.
β-4-GalNT-III	Increased expression in neuroblastoma tumors correlated with favorable histologic profile and early clinical staging.
β-4-GalT-I	High expression correlates to poor survival, enhanced tumor size, increased lymph node metastasis, elevated cancer progression, and enhanced incidence of relapse in pancreatic ductal adenocarcinomas. It predicts prognosis and adjuvant chemotherapy benefits in muscle-invasive bladder cancer. It is a new factor involved in the propagation and maintenance of lung cancer stem cells. Increased expression is a potential independent adverse prognostic factor for survival in patients with non-metastatic clear-cell renal cell carcinoma. It is sufficient to inversely regulate the malignant behaviors of HCC/CRC cells. It may play a vital role in the occurrence of acute myeloid leukemia.
β-4-GalT-III	It contributes to tumorigenic activities by 1-integrin pathway in cervical cancer cells. It promotes cell proliferation and invasion in glioblastoma and might be a potential molecular therapeutic target.
β-4-GalT-IV	It is a critical promoter for hepatocellular carcinoma.
β-3-GalT-V	It is involved in AKT/JNK1/2/MAPK cancer pathways. It binds to CSNK2A1 to regulate its expression in gastric cancer cells. Expression is linked to advanced stages and poor outcomes in hepatocellular carcinoma while promoting aerobic glycolysis. It is a potential therapeutic target for HCC/CRC and promotes aerobic glycolysis. It is critical for tumor growth and lymph node and lung metastasis in PDX mice.
β-4-GalT-V	It may serve as a prognostic biomarker of hepatocellular carcinoma and is involved in the development of multi-drug resistance of human leukemia cells. Expression is increased specifically in colorectal cancer. Overexpression increases growth of astrocytoma cell line. It promotes cell proliferation. It modulates breast cancer stem cells through Wnt/β-catenin signaling pathway and increases stem cell marker ALDH1A1 while promoting production of CD4+. It plays an immunological regulation role and is also concerned with tumor generation, embryonic development, and other malignant diseases.
β-3-GNT-III	Overexpression promotes tumor progression and immunosuppression in pancreatic cancer. Its expression is associated with clinical outcomes in pancreatic carcinomas.
β-III-GNT-V	It is correlated with stem-like phenotype and poor clinical outcome in human gliomas.
β-3-GNT-VI (CORE 3 SYNTHETASE)	It is involved in the development of multi-drug resistance of human leukemia cells. It is involved in FAK/SRC/SHC pathways.

**Table 2:** Some specific oncogenes are frequently observed in different cancers, and their interactions with β-1,4-GalT-V.

Gene	Majorly Affects These	Interactions with β-1,4-GalT-V, and Cancer Pathways
*APC*	Colon, thyroid, stomach, intestine	It is a component of the Wnt/β-catenin signaling pathway that negatively regulates cell growth. It regulates multiple signaling pathways by enhancing GSK-3 activity.
*CDK4*	Melanoma	CDK4/6 inhibition stimulated glycolytic and oxidative metabolism and was associated with an increase in mTORC1 activity. MTOR and MEK inhibitors potently cooperate with CDK4/6 inhibition in eliciting cell cycle exit.
*E-Cadherin*	Stomach	GSK-3 influences epithelial barrier function by regulating Occludin, Claudin-1, and E-cadherin expression. It prevents initial dissociation of epithelial cells from the original tumor mass and loss of cell–cell adhesion and allows cells to invade tissues and migrate to distant sites.
*PTEN*	Hematoma, glioma, uterus	The PTEN tumor suppressor is frequently mutated or deleted in cancer and regulates glucose metabolism through the PI3K-AKT pathway. PTEN accumulation reduced hypoxia induced Akt phosphorylation, thereby repressing glycolysis in cancer cells. Inactivation of PTEN enzymatic activity leads to induction of cell proliferation and inhibition of cell death, causing cancer development and progression.
*TP53 (p53)*	Breast, sarcoma, adrenal, brain	p53 regulates glycolysis, oxidative phosphorylation, lipid metabolism, the PPP, serine synthesis, and nucleotide synthesis. p53 is the most frequently mutated gene in human cancers. p53 has also been reported to suppress glycolysis through regulation of other signaling pathways involved in glycolysis. For instance, activation of the PI3K/AKT signaling drives glycolysis in cancer cells.
*TSC1*, *TSC2*	Hamartoma, kidney	TSC-associated tumors exhibit somatic loss of the second allele of the TSC genes, leading to aberrant activation of the mechanistic target of rapamycin (mTOR) signaling pathway. Activation of mTOR complex 1 (mTORC1) causes addiction to glucose and glutamine in Tsc1^−/−^ or Tsc2^−/−^ MEFs. Blocking of glutamine anaplerosis in combination with glycolytic inhibition causes significant cell death in Tsc2^−/−^.
*WT1*	Wilms’	Mutations in the WT1 gene can affect E-cadherin expression. It can induce epithelial differentiation during mesenchymal–epithelial transition and E-cadherin expression in the embryonic kidney.

**Table 3:** Glycogenes, as seen in other diseases of interest.

Disease	Role of Glycogene
Psoriasis	Zou (2021) suggests that N-glycan markers could serve as diagnostic biomarkers for psoriasis [62]. Sevilla (2019) discusses the role of glucocorticoids (GCs) and the GC-induced Leucine-Zipper (GILZ) in psoriasis treatment [63]. Hannen (2017) highlights dysfunctional skin-derived glucocorticoid synthesis as a pathogenic mechanism in psoriasis [64]. RIPPA (1965) reports increased levels of enzymes related to carbohydrate metabolism in psoriatic skin [65].
Hyperlipidemia	Pietilä (1981) found that hyperlipidemic rabbit serum and its lipid extract impaired the precipitation of glycosaminoglycans [66]. Brownlee (1991) highlighted that hyperglycemia leads to the accumulation of advanced glycosylation end products (AGEPs) in tissues, which can induce abnormalities in protein cross-linking and cell–matrix interactions [67]. Aronson (2002) discussed that nonenzymatic glycosylation of proteins and lipids can interfere with their normal function by disrupting molecular conformation and alter enzymatic activity, leading to hyperglycemia which may promote atherosclerosis [68].

## Abbreviations

ApoE^−/−^	apolipoprotein
ADC	antibody–drug conjugate
ASMC	Airway smooth muscle cell
β-1,4-GalT-V	β-1,4 Galactosyltransferase-V
CAR	Chimeric antigen receptor
CRC	colorectal cancer
CRISPR	Clustered regularly interspaced short palindromic repeats
Cys	Cysteine
D-PDMP	Phenyl-2-decanoylamino-3-N-morpholino-1-propanol
EGF	epidermal growth factor
ELAM-1	endothelial leukocyte adhesion molecule 1
ELISA	enzyme-linked immunosorbent assay
GlcCer	Glucosylceramides
GlcNAc	N-acetylglucosamine
GSL	glycosphingolipids
HUVEC	human umbilical vein endothelial cells
ICAM-1	intercellular adhesion molecule
LacCer	Lactosylceramide
LDL	low-density lipoprotein
MEF	Mouse Embryonic Fibroblasts
NADPH	nicotinamide adenine dinucleotide phosphate
OMIM	Online Mendelian Inheritance in Man
Ox-LDL	oxidized low-density lipoprotein
MAPK	mitogen-activated protein kinases
PDGF	platelet-derived growth factor
PTM	post-translational modification
ROS	reactive oxygen species
SiRNA	small interfering ribonucleic acid
TSC	tuberous sclerosis complex
TM	rransmembrane
TNF-α	Tumor necrosis factor-alpha
TSG-6	Tumor necrosis factor-inducible gene 6
Xyl	Xylose
UDP-galactose	Uridine diphosphate galactose
VCAM-1	vascular cell adhesion molecule-1
VEGF	vascular endothelial growth factor
